# Factors Impacting Early Mortality in Tuberculosis/HIV Patients: Differences between Subjects Naïve to and Previously Started on HAART

**DOI:** 10.1371/journal.pone.0045704

**Published:** 2012-09-25

**Authors:** Carolina Arana Stanis Schmaltz, Guilherme Santoro-Lopes, Maria Cristina Lourenço, Mariza Gonçalves Morgado, Luciane de Souza Velasque, Valéria Cavalcanti Rolla

**Affiliations:** 1 Clinical Research Laboratory on Mycobacteriosis of Instituto de Pesquisa Clínica Evandro Chagas – Fundação Oswaldo Cruz (Fiocruz), Rio de Janeiro, Brazil; 2 Infectious Diseases Clinic, Hospital Universitário Clementino Fraga Filho and Department of Preventive Medicine, Universidade Federal do Rio de Janeiro, Rio de Janeiro, Brazil; 3 Bacteriology Laboratory, Instituto de Pesquisa Clínica Evandro Chagas, Fiocruz, Rio de Janeiro, Brazil; 4 Immunology Laboratory, Instituto de Pesquisa Clinica Evandro Chagas, Fiocruz, Rio de Janeiro, Brazil; 5 Department of Mathmatics and Statistics, Universidade Federal do Estado do Rio de Janeiro, Rio de Janeiro, Brazil; McGill University, Canada

## Abstract

**Background:**

Mortality among patients with tuberculosis (TB)/HIV is highest during the first few months of antituberculous therapy. The objective of this study was to assess the factors associated with early mortality among TB/HIV patients and whether these factors are similar for HAART naïve and those with prior HAART initiation.

**Methods:**

Prospective cohort study including HIV patients with tuberculosis confirmed by culture, cared for at a referral center in Rio de Janeiro, Brazil. Multivariable Cox analysis was used to assess predictors of mortality within 3 months of antituberculous therapy.

**Results:**

Among 227 patients included, 90 (40%) started HAART before TB diagnosis. The median time to TB diagnosis after ARV initiation was 5.9 months (interquartile range [IQR] 3.0–8.9 months). Fourteen patients (6%) died within the first 3 months. Mortality was not different between patients previously started on HAART and those who were naïve to it. In the overall adjusted analysis, HAART use during TB treatment (hazard ratio [HR] = 0.21, 95% confidential interval [CI] = 0.06–0.72) and CD4 lymphocyte count >100 cells/mm3 (HR = 0.21, 95% CI = 0.04–0.99) were associated with lower mortality, while subjects with unknown baseline CD4 lymphocyte count (HR = 9.39, 95% CI = 2.56–34.5) had higher mortality. In subgroup analysis, among HAART naïve subjects, disseminated TB (HR = 5.32, 95% CI = 1.09–25.8) and unknown baseline CD4 lymphocyte count (HR = 13.2, 95% CI = 2.71–64.5) were associated with significantly higher mortality, while HAART (HR = 0.14, 95% CI = 0.03–0.69) predicted a better outcome. Among subjects previously started on HAART, mortality was significantly associated with duration of TB symptoms >120 days (HR = 6.15, 95% CI = 1.15–32.9).

**Conclusions:**

Predictors of early mortality among TB/HIV patients may vary according to the timing of HAART initiation. Among HAART naïve patients, mortality was influenced by baseline clinical severity, HAART use and, possibly, the quality of care preceding TB diagnosis. For patients with prior HAART initiation, longer delays in TB diagnosis predicted a significantly higher mortality.

## Introduction

Tuberculosis (TB) is an important cause of death worldwide, especially in resource-limited countries, where the intersection with HIV pandemics has led to a marked increase in its prevalence. In the last fifteen years, a considerable international effort has been developed, under the coordination of the World Health Organization, to achieve global improvement of TB control. The Global Plan to Stop TB set in 2006 [1] includes in its goals a reduction in TB mortality rates by 2015 to half their level in 1990. Among HIV negative subjects, there is a current trend for a reduction in the TB–attributable mortality rate that predicts it will be halved by 2015 [2]. Nevertheless, among HIV positive subjects, who comprise about 12% of all TB cases around the world, the decline in mortality has been less pronounced and, thus, the achievement of that goal is unlikely. Among TB/HIV patients who start antituberculous therapy, the mortality rate is highest within the first three months after the diagnosis of TB [3]–[6]. Most deaths attributable to TB occur in this early period, whereas later mortality is more probably related to other causes and, therefore, less amenable to interventions specifically aimed at improving TB control and outcome among HIV infected subjects. Factors that possibly influence early mortality among TB/HIV patients include delayed presentation with advanced TB and AIDS; late diagnosis of TB within health services; the simultaneous occurrence of other life-threatening HIV-related complications; lack of access to antiretroviral therapy, multi-drug resistant TB infection and immune reconstitution [6]–[11]. However, to our knowledge, no study has specifically assessed the predictors of early mortality among HIV subjects who recently started antituberculous therapy. Additionally, the available data on early mortality among TB/HIV subjects come from studies that excluded patients who had previously begun antiretroviral therapy. In countries where antiretroviral therapy has been widely available for a long time, such as in Brazil, there is an increasing proportion of HIV patients who start antituberculous therapy already on HAART [12] and it is plausible that factors influencing early mortality among them may not be the same as observed among HAART naïve subjects.

In this study, we analyze data from a cohort of TB/HIV patients followed at a referral center in Rio de Janeiro, Brazil, that includes subjects who already begun anti-retroviral therapy as those who have never used it. The aims of the study were to examine, in this setting, which factors were associated with early mortality among TB/HIV patients starting antituberculous therapy, and to assess if these predictors of mortality were similar for HAART naïve and with prior HAART initiation patients.

## Methods

### Ethics Statement

Patients were included in the study after signing a written informed consent form.The Committee on Ethics in Research of IPEC approved the study.

### Population and Design

This study analyzes data from an ongoing prospective cohort study [13] carried out at the Instituto de Pesquisa Clínica Evandro Chagas (IPEC, Fiocruz). IPEC is a reference hospital for infectious diseases inside the campus of Fiocruz and care for TB and HIV patients in two integrated programs.

HIV patients, aged 18 years or older, with TB diagnosis confirmed by culture, who started antituberculous therapy from April 14^th^, 2000 to January 22^nd^, 2010 were included. Patients were followed up until August 23^rd^, 2010.

### Data Collection and Follow-up

Follow-up start was determined by the date of the first prescription of antituberculous therapy, what was done at baseline visit. Data collected in this baseline visit included sex, age, monthly income, education, alcohol abuse, drug abuse, weight loss, clinical presentation of TB, and the results of the following laboratorial tests: microscopic examination of sputum; culture of sputum, blood and other clinical specimens for mycobacteria and fungal pathogens; serologic tests for *Histoplasmacapsulatum*, *Paracoccidioides brasiliensis*, viral hepatitis, toxoplasmosis and syphilis; latex assay for cryptococcal antigen; blood cell counts; serum levels of creatinine, liver enzymes and albumin; date of first positive HIV serology; history of opportunistic diseases, current and previous antiretroviral (ARV) therapy regimens. The most recent results of CD4 cell count and HIV viral load within the preceding six months were also noted. For HAART naïve patients with no previous measurement of CD4 cells and HIV viral load, the baseline value was defined as the first measurement obtained after inclusion in the study, but before the start of HAART.

Follow-up visits in the outpatient clinic were scheduled on day 15, 30 and 60 after the initial visit and bimonthly thereafter. Data recorded in these visits included: clinical manifestations associated with TB or HIV infection, the occurrence of immune reconstitution syndrome (IRIS), adverse events related to HAART or antituberculous therapy. All patients who required hospitalization during the studied period were admitted to IPEC.

### Microbiologic Methods

Ziehl-Neelsen staining was used for the detection of acid-fast bacilli in sputum smears and biopsy specimens. Sputum and biopsy samples were cultured in Löwenstein-Jensen medium. Culture of blood specimens used the lysis-centrifugation method. Drug susceptibility tests for rifampicin, isoniazid, pyrazinamide, ethambutol, streptomycin and ethionamide were performed by the agar proportion method.

### Outcome

The primary study outcome was death during the first 3 months of TB treatment.

### Definitions

The clinical presentation of TB was classified as pleural-pulmonary (when restricted to the lungs and/or pleura), extra-pulmonary localized (when just one extra-pulmonary site was affected), or disseminated. *M. tuberculosis* isolates resistant to at least rifampicin and isoniazid were defined as multi-drug resistant.

IRIS was defined as a documented worsening of signs or symptoms of TB during appropriate antituberculous treatment and following the initiation of antiretroviral therapy, not explained by any other disease or by an adverse effect of drug therapy.

The cause of death was determined afterthorough review of relevant clinical, microbiological and pathological data of each deceased patient. All deaths occurred while patients were hospitalized at IPEC.

### Antituberculous and Antiretroviral Therapy

Antiretroviral therapy was offered according to contemporary Brazilian National Guidelines that were periodically updated. Cotrimoxazole prophylaxis was routinely prescribed.

The first line antituberculous regimen during most of the study period was the combination of rifampicin, isoniazid and pyrazinamide during the two initial months (“intensive phase”), followed by rifampicin plus isoniazid during four months (“continuation phase”) except in cases with central nervous system disease when the continuation phase was extended to 7 months. From July 2009 on, ethambutol was added to the intensive phase regimen following a new recommendation from the Brazilian Ministry of Health. TB treatment was adjusted in cases of severe adverse events, drug resistance and HAART regimens that precluded the use of rifampicin.

### Statistical Analysis

Chi-square and Fisher’s exact test were used to compare the distribution of categorical variables. The distribution of continuous variables was compared by the Mann-Whitney test. The influence of studied variables on the risk of TB-related mortality was assessed in univariate and multivariate Cox proportional hazards models. In these analyses patients who did not die from TB had their follow-up censored on the 90^th^ day after the start of antituberculous therapy. In all analyses, the use of HAART during antituberculous therapy was modeled as a time-dependent variable with an intention-to-treat approach. Variables that occurred with a frequency ≥10% were selected for the multivariate survival analysis if associated with a p-value ≤0.20 in univariate analysis. Additionally, variables that were associated with the baseline exposure to anti-retroviral therapy (p value ≤0.20) were selected for a subsequent stratified survival analysis. All adjusted models were obtained with backward stepwise selection. Variables associated with p value ≤0.10 were retained in these models. A statistically significant association with the studied outcome was defined by a p-value ≤0.05. All p-values are two-tailed and were calculated with the Wald test. These analyses were performed in R for Windows v. 2.5.1(R Foundation for Statistical Computing).

## Results

**Figure 1:**
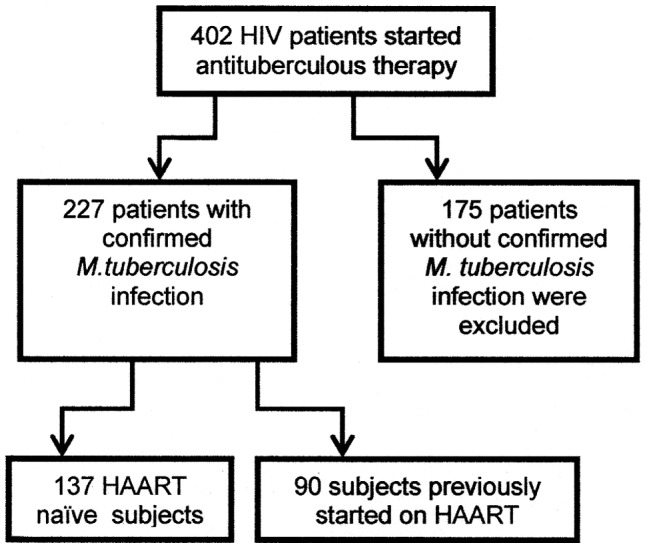
Profile of the patients included in the study.

Among 402 TB/HIV patients that started antituberculous treatment during the analyzed period, 227 subjects (56.5%) with a diagnosis of *M. tuberculosis* infection confirmed by culture were included in the study (Figure 1). Ninety subjects (40%) started HAART before the diagnosis of TB with a median time since the initial prescription of anti-retroviral therapy of 5.9 months (interquartile range [IQR]: 3.0–8.9 months). Among the 137 HAART naïve patients, 103 started HAART after a median of 34 days (IQR: 27–60 days) on antituberculous therapy. The most frequent HAART regimen was the combination of 2 nucleoside reverse transcriptase inhibitors with efavirenz (139 patients). The distribution of other baseline and follow-up variables is described in Table 1. Results of CD4 cell count and HIV viral load preceding the start of antituberculous therapy were available for 111 and 93 patients, respectively. The median time lag between these measurements and the start of antituberculous therapy was 50 days (IQR: 19–115 days) for CD4 cell count and 51.5 days (IQR: 20–127 days) for HIV viral load. In 84 HAART naïve patients with a recent diagnosis of HIV infection, the first measurement of both variables was obtained a median 28.5 days (IQR: 14–41 days) after the start of antituberculous therapy. In all these 84 cases, these measurements were performed before the start of HAART.

**Table 1:** Distribution of Baseline and Follow-up Variables.

Variable	N (%)
White race	107 (47)
Age >40	74 (33)
Male sex	162 (71)
Men who have sex with men	66 (29)
Alcohol abuse	54 (24)
Intravenous drug use	12 (5)
Use of other illicit drugs	64 (28)
School education >8 years	88 (39)
Monthly income ≤ U$500.00	161 (71)
Time since HIV infection diagnosis (months)^a^	16 (0–78) 78)*median (interquartile range);*
HAART naïve	137 (60)
TB as criteria for AIDS diagnosis	97 (43)
TB clinical presentation	
Pleural-pulmonary	119 (52)
Extra-pulmonary, localized	32 (14)
Disseminated	76 (34)
Weight loss >10%	153 (65)
Positive sputum smear	146 (64)
Hemoglobin ≥10g%	113 (50)
Serum albumin ≥3g%^b^	95 (42)
Duration of symptoms before antituberculous treatment^a^	90 (45–120)
Initial CD4 cell count (cells/mm^3^)a,c	168 (79–291)
Initial viral load (log)a,d	4.9 (3.9–5.5)
Resistance to one antituberculous drug	23 (10)
Multi-drug resistance	8 (4)
Susceptible TB infection treated with rifampicin	185 (82)
IRIS	14 (6)
Used HAART during antituberculous treatment	193 (85)

TB = tuberculosis; HAART = highly active anti-retroviral therapy; IRIS = immune reconstitution inflammatory syndrome;

a median (interquartile range);

b missing data for 37 (16%) patients;

c missing data for 32 (14%) patients;

d missing data for 50 (22%) patients.

There were 14 (6%) deaths, all related to tuberculosis. The mortality rate during the first 3 months of antituberculous therapy was 25.9/100 patient-years. There were no losses to follow-up during the analyzed period. All 20 cases (9%) of therapy default observed in this cohort occurred after the 3 month of antituberculous treatment.

In univariate Cox analyses (Table 2), hemoglobin ≥10 g% (p = 0.02), serum albumin ≥3 g% (p = 0.04), CD4 lymphocyte count ≥100 cells/mm^3^ (p = 0.04) and HAART use during TB treatment (p = 0.01) were associated with a significant lower risk of early mortality. On the other hand, patients with missing baseline CD4 cell count (p<0.001), disseminated TB (p = 0.01) and higher viral load (p = 0.03) had a significantly worse outcome. There was no significant difference on mortality between HAART naïve subjects and patients who previously begun antiretroviral therapy (p = 0.99).

**Table 2:** Univariate Cox Analysis of Predictors of Early Mortality.

Variable	Deaths(total = 14)n (%)	Survivors(total = 213)n (%)	Hazard ratio(95% CI)	p
White race	7 (50)	100 (47)	1.13 (0.39–3.22)	0.82
Age >40	6 (43)	68 (32)	1.52 (0.53–4.35)	0.43
Male sex	12 (86)	150 (70)	2.53 (0.56–11.5)	0.23
Men who have sex with men	4 (29)	62 (29)	1.07 (0.33–3.48)	0.90
Alcohol abuse	3 (22)	51 (24)	0.97 (0.26–3.53)	0.96
Intravenous drug use	1 (7)	11 (5)	1.31 (0.18–9.53)	0.79
Other illicit drugs use	3 (22)	61 (29)	0.73 (0.20–2.62)	0.63
School education >8 years	6 (43)	82 (39)	1.19 (0.41–3.40)	0.75
Monthly income ≤ U$500.00	9 (64)	152 (71)	0.87 (0.27–2.78)	0.82
Time since HIV diagnosis (for each month)^a^	3.5	17.0	1.00 (0.99–1.01)	0.97
HAART naïve	8 (57)	129 (61)	0.99 (0.30–3.26)	0.99
TB as criteria for AIDS diagnosis	8 (57)	89 (42)	2.26 (0.66–7.74)	0.20
Disseminated TB	9 (64)	67 (32)	4.02 (1.37–11.8)	0.01
Weight loss >10%	7 (50)	146 (69)	0.58 (0.18–1.88)	0.36
Positive smear	11 (79)	135 (63)	3.13 (0.67–14.7)	0.15
Hemoglobin ≥10g%	3 (21)	110 (52)	0.23 (0.06–0.81)	0.02
Serum albumin ≥3g%^b^	3 (21)	92 (51)	0.26 (0.07–0.96)	0.04
TB symptoms for >120 days	6 (43)	66 (31)	1.65 (0.57–4.8)	0.36
CD4 cell count >100 cells/mm^3c^	3 (21)	126 (56)	0.27 (0.08–0.96)	0.04
Unknown CD4 cell count	7 (50)	25 (12)	6.80 (2.33–20.0)	<0.001
Viral load (for each log/mL)^d^	5.22	4.89	9.40 (1.29–68.7)	0.03
Resistance to one anti-TB drug	0	23 (11)	Undefined	0.07
Multi-drug resistance	0	8 (4)	Undefined	0.30
Susceptible TB treated with rifampin	11 (79)	174 (82)	0.85 (0.23–3.07)	0.80
IRIS	2 (14)	12 (6)	2.73 (0.61–12.0)	0.19
HAART^e^	7 (50)	186 (87)	0.23 (0.07–0.76)	0.01

TB = tuberculosis; CI = confidence interval; HAART = highly active anti-retroviral therapy; IRIS = immune reconstitution inflammatory syndrome;

a median;

b missing data for 37 (16%) patients;

c missing data for 32 (14%) patients;

d missing data for 50 (22%) patients;

e time-dependent variable.

In the overall multivariate analysis (Table 3), HAART use during TB treatment (p = 0.01) and CD4 lymphocyte count ≥100 cells/mm^3^ (p = 0.05) were associated with significantly lower mortality, while subjects with unknown baseline CD4 cell count (p<0.001) had a significantly higher mortality.

**Table 3:** Variables Associated with Early Tuberculosis-Related Mortality in Multivariate Analysis.

Variable	Hazard ratio	95% CI	P
HAART^a^	0.21	0.06–0.72	0.01
Baseline CD4 cell count^b^			
≥100 cells/mm^3^	0.21	0.04–0.99	0.05
Unknown	9.39	2.56–34.5	<0.001

CI = confidence interval, HAART = highly active anti-retroviral therapy;

a time-dependent variable;

b reference category: CD4 cell count <100 cells/mm^3^.

Subjects with prior HAART initiation differed significantly from HAART naïve patients with regard to several aspects (Table 4). The later were younger (p<0.001), had a more recent diagnosis of HIV infection (p<0.001), more frequently presented with TB as the AIDS-defining condition (p<0.001), had disseminated disease (p<0.001), weight loss ≥10% (p = 0.003), a higher baseline viral load (p<0.001) and more frequently developed IRIS (p = 0.05).

**Table 4:** Distribution of Variables According to HAART Status at Baseline.

Variable	HAART naïve(total = 137)n(%)	Prior HAART initiation (total = 90)n (%)	P
White race	63 (48)	44 (49)	0.74
Age >40	32 (23)	42 (47)	<0.001
Male sex	102 (75)	60 (67)	0.20
Men who have sex with men	41 (30)	25 (28)	0.71
Alcohol abuse	35 (26)	19 (21)	0.42
Intravenous drug use	7 (5)	5 (6)	1.0
Other illicit drugs use	38 (28)	26 (29)	0.89
School education >8 years	56 (41)	32 (36)	0.40
Monthly income ≤ U$500.00	102 (75)	59 (66)	0.18
Time since HIV infection diagnosis (months)	1(0–12)^a^	70(36–106)^a^	<0.001
TB as criteria for AIDS diagnosis	97 (100)	0	<0.001
Disseminated TB	58 (42)	18 (20)	<0.001
Weight loss >10%	104 (76)	49 (54)	0.003
Positive smear	94 (69)	52 (58)	0.12
Hemoglobin ≥10g%	66 (51)	47 (55)	0.48
Serum albumin ≥3g%^b^	52 (44)	43 (57)	0.08
TB symptoms for >120 days	48 (35)	24 (27)	0.19
CD4 cell count ≥100 cells/mm^3^	71 (65)	58 (73)	0.25
Unknown CD4 cell count	22 (16)	10 (11)	0.30
Viral load (median log/mL)^c^	5.10 (4.57–5.61)^a^	4.38 (2.14–5.11)^a^	<0.001
Resistance to one anti-TB drug	14 (10)	9 (10)	0.98
Multi-drug resistance	7 (5)	1 (1)	0.15
Susceptible TB treated with rifampin	116 (85)	69 (77)	0.13
IRIS	12 (9)	2 (2)	0.05

HAART = highly active anti-retroviral therapy; TB = tuberculosis; IRIS = immune reconstitution inflammatory syndrome;

a median (interquartile range).

b missing data for 37 (16%) patients.

c missing data for 50 (22%) patients.

Subsequently, multivariate survival analyses stratified according to the baseline exposure to HAART were performed. The final adjusted models are shown in Table 5. Among subjects previously started on HAART, the presence of symptoms lasting longer than 120 days before TB diagnosis (p = 0.03) was significantly associated with higher mortality. There was also an association, with borderline statistical significance, between mortality and a baseline viral load >5 log/ml (p = 0.08). Among HAART naïve subjects, mortality was significantly higher for those with unknown baseline CD4 cell count (p = 0.001) and who had disseminated TB (p = 0.04), whereas HAART use during TB treatment (p = 0.02) was associated with longer survival. Patients with baseline albumin ≥3 g% also tended to have a better outcome (p = 0.07).

**Table 5:** Multivariate Analyses of Factors Associated with Early Tuberculosis-Related Mortality in Subgroups Defined According to the Baseline Status of HAART use.

Subgroup	Variable	Hazard ratio	95% CI	P
**HAART started previously to TB diagnosis**	TB symptoms for >120 days	6.15	1.15–32.9	0.03
	Viral load >5 log/ml	6.85	0.78–60.0	0.08
**HAART naïve**	Disseminated TB	5.32	1.09–25.8	0.04
	Albumin ≥3g/dl	0.13	0.02–1.16	0.07
	Unknown CD4 cell count	13.2	2.71–64.5	0.001
	HAART	0.14	0.03–0.69	0.02

CI = confidence interval, HAART = highly active anti-retroviral therapy, TB = tuberculosis.

## Discussion

Most data on the rate and predictors of mortality in TB/HIV patients were obtained in patients who, at the time of start of antituberculous therapy, were naïve to HAART [6], [14], [15]. However, the incidence of TB among HIV patients on antiretroviral therapy is still increased as compared to the general population, even after long term of HAART use [16] and, thus, it is predictable that in areas where HAART has been widely available for a long period, as in Brazil, a considerable proportion of patients presenting with TB will not be naïve to antiretroviral therapy.

In this cohort of TB/HIV patients, cared for at a referral center in a middle-income country, 40% of the patients had previously started antiretroviral therapy, a proportion that is similar to that reported in a population-based study in Denmark [12]. Most of the HAART naïve subjects began antiretroviral therapy within 8 weeks after the start of antituberculous therapy. In spite of the high proportion of patients that received antiretroviral therapy during the initial phase of antituberculous treatment, the rate of early mortality was still considerably high and comparable to those reported from studies conducted in other resource-limited settings, in which only HAART naïve patients were included [6], [17]. Mortality was similar for subjects that started HAART before TB diagnosis and HAART naïve patients. This finding is probably related with the fact that in most of previously HAART experienced patients in this cohort, the occurrence of TB was associated with failure of the current antiretroviral regimen.

The analysis of predictors of mortality in the overall study population showed that early mortality was higher among patients not treated with HAART and whose baseline CD4 lymphocyte count was low or unknown. These findings are consistent with the results of other reports [15]–[23].

HAART use during TB treatment was associated with an estimated 80% reduction in early mortality. Several studies have shown that the start of HAART during the course of tuberculosis reduces mortality [15], [18]–[25]. Recent trials have demonstrated a beneficial impact of HAART early start (between 2 to 4 weeks after the initiation of antituberculous therapy) among TB/HIV patients with advanced immunodeficiency after a median follow-up of 18 to 25 months. [14], [23], [26]. Other observational studies have already detected an improve on survival among TB/HIV patients using HAART with less advanced immunodeficiency [6], [15]. The reason for these different findings is not clear and might be related to differences in the characteristics of the population included in each study. Nevertheless, we must recognize that, given the observational design of the present study, it is not possible to rule out the possibility of selection bias or residual confounding.

The finding that HIV patients who started antituberculous therapy with unknown CD4 cell count had higher mortality has been described elsewhere and it suggests that patients who lacked a baseline CD4 cell count, actually, presented advanced immunodeficiency [15]. Such association may be a result of late diagnosis of HIV or of underutilization of health care by subjects with known HIV infection [27]. This finding also underscores a relevant logistical challenge for the adequate care of TB/HIV patients. As the results of recent trials suggested that the decision to start HAART early in the intensive phase of antituberculous therapy may depend on CD4 cell count measurement [23], [26], lack of access to this laboratorial test may unnecessarily delay the start of HAART in subjects in whom it is most needed. In such cases, clinical suspicion of advanced immunodeficiency should prompt the early start of HAART.

The results of the subsequent subgroup analysis were consistent with the hypothesis that predictors of mortality among TB/HIV patients may vary according to their antiretroviral therapy status at baseline.

Among patients who started HAART prior to TB diagnosis, longer duration of TB symptoms before the start of antituberculous therapy was associated with a significant higher risk of early mortality. Mortality also tended to be higher among patients with HIV viral load >5.0 log-copies/mL. The borderline statistical significance associated with this variable was probably related to the limited statistical power in the adjusted stratified analysis.

The diagnosis of TB in HIV infected subjects represents a major challenge given the lower yield in this population of conventional diagnostic methods, such as sputum smear, chest X-ray and the long time to isolate *M. tuberculosis* in solid medium culture. In this study, about 30% of the patients had TB symptoms for ≥120 days before this diagnosis was established. However, there was no significant difference regarding this variable between the studied subgroups. The reason for this differential impact of delayin TB diagnosis on survival among patients with prior HAART initiation as compared to HAART naive subjects is not clear from our data. It could be caused by a lack of sureness of TB diagnosis among these patients before culture results were available. Initiating TB treatment in these patients is generally associated with a necessity to change the antiretroviral regimen because of interaction with anti-TB drugs, what sometimes is difficult to implement because of the lack of an alternative effective regimen. Nonetheless, this finding suggest that the survival benefit to be derived from the implementation of faster and more sensitive diagnostic methods for TB, including urinary lipoarabinomannan detection, simplified nuclear acid amplification tests and improved culture-based systems [28] will probably be more pronounced in this subgroup of patients.

The observed trend for an association between poor virologic control and higher mortality probably reflects the lack of effectiveness of the antiretroviral regimen used at baseline, either because of no adherence at all to it or because of the accumulation of resistance mutations as a result of past suboptimal adherence. In some of these cases, survival may have been influenced by the fact that there was no effective antiretroviral regimen that could be used concomitantly to rifampicin at the time of TB diagnosis.

In the subgroup of HAART naïve patients, the start of antiretroviral therapy was associated with a significantly better outcome. Baseline serum albumin levels ≥3 g% also tended to be associated with lower mortality. On the other hand, disseminated TB and the lack of any CD4 cell count measurement within 6 months before the diagnosis of TB were significantly associated with a poorer short term outcome. These data suggest that late presentation and underutilization of medical resources may limit the benefit that early antiretroviral therapy can bring to this subset of patients in whom the diagnosis of HIV infection was frequently unknown before the appearance of clinical manifestations of TB.

In addition to its observational design, this report has several other limitations. It is a single site study performed in a center with integrated TB and HIV services, highly trained staff and good laboratorial support, characteristics that may not be representative of the average standard of care in other resource limited areas. Finally, this is an exploratory analysis and their results must be interpreted with cautious. As there were only 14 deaths during the studied period, the statistical power of Cox analysis is low and, therefore, other possible predictors of death could have been missed, especially in the stratified analysis.

In conclusion, the rate of early deaths among HIV infected subjects presenting with TB remains high in resource limited countries, even in areas, such as Brazil, where free access to antiretroviral therapy has been provided for more than a decade. The short term outcome for patients with prior HAART initiation was similar to that observed among HAART naïve TB/HIV patients. Predictors of early mortality seem to be different between these two subgroups of patients. Among HAART naïve patients, mortality was influenced by baseline signs of TB/HIV severity, HAART use during TB treatment and, possibly, the quality of care preceding TB diagnosis. For patients who had previously started HAART, longer delays in TB diagnosis and poor control of viral replication predicted higher mortality. Our results suggest that, in this setting, further reduction on TB-related mortality may depend on earlier suspicion of TB diagnosis, access to faster diagnostic methods for TB, especially among patients already on antiretroviral therapy.
